# Risk factors for pre-term birth in a Canadian cohort of HIV-positive women: role of ritonavir boosting?

**DOI:** 10.7448/IAS.18.1.19933

**Published:** 2015-06-05

**Authors:** Fatima Kakkar, Isabelle Boucoiran, Valerie Lamarre, Thierry Ducruet, Devendra Amre, Hugo Soudeyns, Normand Lapointe, Marc Boucher

**Affiliations:** 1 Division of Infectious Diseases, CHU Sainte-Justine, Montréal, Canada;; 2 Department of Pediatrics, Faculty of Medicine, Université de Montréal, Montreal, Canada; 3 Centre maternel et infantile sur le SIDA, CHU Sainte-Justine, Montreal, Canada; 4 Department of Obstetrics and Gynecology, Université de Montréal, CHU Sainte-Justine, Montreal, Canada; 5 Unité de recherche clinique appliquée, CHU Sainte-Justine, Montréal, Canada; 6 Centre de recherche du CHU Sainte-Justine, Montréal, Canada; 7 Department of Microbiology, Infectious Diseases and Immunology, Faculty of Medicine, Université de Montréal, Montréal, Canada

**Keywords:** pre-term birth, mother-to-child prevention, HIV transmission, antiretroviral drugs, protease inhibitors

## Abstract

**Background:**

The risk of pre-term birth (PTB) associated with the use of protease inhibitors (PIs) during pregnancy remains a subject of debate. Recent data suggest that ritonavir boosting of PIs may play a specific role in the initiation of PTB, through an effect on the maternal–fetal adrenal axis. The primary objective of this study is to compare the risk of PTB among women treated with boosted PI versus non-boosted PIs during pregnancy.

**Methods:**

Between 1988 and 2011, 705 HIV-positive women were enrolled into the Centre Maternel et Infantile sur le SIDA mother–infant cohort at Centre Hospitalier Universitaire Sainte-Justine in Montreal, Canada. Inclusion criteria for the study were: 1) attendance at a minimum of two antenatal obstetric visits and 2) singleton live birth, at 24 weeks gestational or older. The association between PTB (defined as delivery at <37 weeks gestational age), antiretroviral drug exposure and maternal risk factors was assessed retrospectively using logistic regression.

**Results:**

A total of 525 mother–infant pairs were included in the analysis. Among them, PI-based combination anti-retroviral therapy was used in 37.4%, boosted PI based in 24.4%, non-nucleoside reverse transcriptase inhibitor (NNRTI) or nucleoside reverse transcriptase inhibitor based in 28.1%, and no treatment was given in 10.0% of cases. Overall, 13.5% of women experienced PTB. Among women treated with antiretroviral therapy, the risk of PTB was significantly higher among women who received boosted versus non-boosted PI (OR 2.01, 95% CI 1.02–3.97). This remained significant after adjusting for maternal age, delivery CD4 count, hepatitis C co-infection, history of previous PTB, and parity (aOR 2.17, 95% CI 1.05–4.51). There was no increased risk of PTB with the use of unboosted PIs as compared to NNRTI- or NRTI-based regimens.

**Conclusion:**

While previous studies on the association between PTB and PI use have generally considered all PIs the same, our results would indicate a possible role of ritonavir boosting as a risk factor for PTB. Further work is needed to understand the pathophysiologic mechanisms involved, and to identify the safest ARV regimens to be used in pregnancy.

## Introduction

The risk of pre-term birth (PTB) associated with the use of protease inhibitors (PIs) in pregnancy remains the subject of intense debate. While a number of studies from Europe have found an increased risk of PTB associated with PI use [1–4], until recently these findings have not been replicated in North American cohort studies [5–9], or in a meta-analysis on the subject [10]. The conflicting reports and the difficulties in establishing such an association are in part due to the heterogeneity of comparison groups (comparing PI use to no treatment and or multiple different treatments), heterogeneity of populations studied and multiple confounders that have been inconsistently adjusted for in previous studies [11–13]. As a result, the question as to a link on PI use and prematurity remains unresolved, and in the absence of definitive evidence to support or refute this potential association, North American guidelines continue to recommend PI-based combination antiretroviral therapy (cART) as the preferred treatment of HIV-1-positive women during pregnancy [14].

The use of PIs during pregnancy has changed significantly over time, as newer drugs were approved for use in HIV-positive pregnant women, and data became available on their safety and efficacy in preventing vertical transmission. Nelfinavir (NFV) was the preferred PI in pregnancy until 2006, due to its tolerability and low side-effect profile [15]. This changed abruptly in 2007 after NFV was recalled from both United States and European markets for concerns regarding the presence of a potential carcinogenic element, ethyl methanesulfonate (EMS) in its formulation [16]. Ritonavir-boosted lopinavir (LPV/r) then became the preferred PI used in pregnancy [17], and NFV, even after safety issues regarding EMS were resolved, became an “alternative” choice to be used under special circumstances [18]. These changes were reflected in prescribing patterns in both North America and Europe, with an increased use of LPV-r as the preferred PI for the treatment of HIV-positive pregnant women after 2007 [19].

Previous studies on PTB have generally considered PIs as a single group for comparison against no treatment, non-nucleoside reverse transcriptase inhibitor (NNRTI) or nucleoside reverse transcriptase inhibitor (NRTI) based regimens, not distinguishing whether they were used with or without ritonavir (RTV) boosting [20]. However, RTV, used individually or as a boosting agent, has its own unique side-effect profile. It is a potent inhibitor of cytochrome CYP3A4, which is implicated in the regulation of the adrenal axis [21]. Fetal signals coming from the hypothalamic–pituitary–adrenal axis are suspected to play a fundamental role in the initiation of spontaneous labour, through elevations in cortisol levels associated with progesterone withdrawal and prostaglandin formation [22]. Transient adrenal dysfunction has been reported in neonates exposed to LPV-r *in utero* and after birth [23]. Thus, given RTV's potential impact on PTB through both the fetal and maternal adrenal systems, and the shift to RTV-boosted PIs in pregnancy after 2007, the primary objective of this study was to assess the risk of PTB associated with RTV boosting in pregnancy, in a single centre North American setting.

## Methods

### Study population

This was a retrospective study using data from the Centre Maternel et Infantile sur le SIDA (CMIS) mother–child cohort. The CMIS cohort was established in 1988 to follow all HIV-positive pregnant women presenting to Centre Hospitalier Universitaire (CHU) Sainte-Justine, a tertiary referral centre in the city of Montreal, and the largest maternal–child health centre in the province of Quebec, Canada. HIV-positive pregnant women who consented to the cohort were prospectively followed during pregnancy, and their infants until age 18, to monitor for effects from *in utero* antiretroviral (ARV) drug exposure. The study was approved by the ethics committee of the CHU Sainte-Justine research centre.

Inclusion criteria for this study were: 1) attendance for at least two antenatal obstetric visits and 2) singleton live births, at 24 weeks of gestational age or older. Two antenatal visits (at minimum) were required to ensure that the patients included in the study were those engaged in care at CHU Sainte-Justine, thereby excluding those who presented only at delivery. ARV use during pregnancy was categorized into groups defined by the last ARV regimen used during pregnancy, as follows: 1) RTV-boosted PI-based regimen: LPV/r, atazanavir (ATV/r), tipranivir (TPV/r) or fosamprenavir (FPV/r) with two NRTIs; 2) unboosted PI-based regimen: NFV, indinavir (IDV) or saquinavir (SQV) with two NRTIs; 3) NNRTI based (nevirapine with 2 NRTIs); 4) NRTI based: zidovudine (AZT) monotherapy, AZT and lamividune (3TC) or triple combination NRTI; and 5) no treatment (no ARVs used at any time during pregnancy). ARV therapy during pregnancy was initiated after completion of the first trimester in treatment naïve patients, and adjusted for treatment-experienced patients according to standard North American guidelines. Additional variables studied included maternal CD4 count nearest to the time of delivery (measured by flow cytometry, up to 1 week before or 1 week after delivery), hepatitis C status (determined by hepatitis C antibody testing during pregnancy) and ethnicity, characterized as Caucasian, Black or other (mixed, First Nations, Latino or Asian).

### Statistical analysis

The association between maternal risk factors and PTB was assessed using odds ratios, and multivariable logistic regression was used to adjust for potential confounders identified in the study population and from the literature (maternal age, delivery CD4 count, parity, hepatitis C co-infection and ethnicity). PTB was defined as delivery at less than 37 completed weeks gestational age, determined by best estimates (self-reported date of last menstrual period, ultrasound-data). The multivariable analysis was restricted to only those women with complete data on all variables assessed. Goodness of fit of the final model was assessed using likelihood ratio (LR), and a generalized estimating equation (GEE) was applied to account for repeat pregnancies. All statistical tests were 2-sided and significance was set at *p*<0.05. The analysis was conducted using SAS statistical software, version 9.3 (SAS Institute, Cary, NC).

## Results

Between 1988 and 2011, 705 women were enrolled in the CMIS cohort, among whom 589 met the study's inclusion criteria (19 were excluded due to twin gestation, 28 due to therapeutic or spontaneous abortion, and 69 due to only one visit on record). Overall, during the entire study period, unboosted PI-based cART was used in 220 (37.4%) women, RTV-boosted PI-based cART in 144 (24.4%), NNRTI- or NRTI-based cART (other) in 166 (28.1%), while 59 (10.0%) women received no treatment. Among boosted PIs, the most commonly used was LPV/r (81.2%), followed by ATV/r (13.4%), FPV/r (2.7%) and TPV/r (2.7%). Among unboosted PIs, the most commonly used was NFV (82.4%), followed by IDV (9.2%) and SQV (8.4%). The most common NRTI combinations used as a backbone regimen included AZT/3TC (63.0%), followed by abacavir (ABC)/3TC (4.9%), tenofovir (TDF)/emtricitrabine (FTC) (2.3%), stavudine (d4T) based (d4T/3TC or D4T/TDF) (2.3%) and didanosine based (ddI/3TC, ddI/AZT, ddI/3TC or ddI/TDF) (1.6%) and other (0.8%). A single NRTI (AZT) was used in 15.1% of women, while 10% received no treatment.

Characteristics of the women according to the type of ARV therapy received are summarized in Table 1.

**Table 1:** Maternal characteristics by treatment group

Maternal characteristics	*N*	Boosted PI	Unboosted PI	Other	None	*p* ^a^	*p* ^b^
Year of delivery							
1988–1996	107	0 (0)	0 (0)	59 (35.5)	48 (81.4)	<0.001	<0.001
1997–2006	318	18 (12.5)	196 (89.1)	97 (58.4)	7 (11.9)		
2007–2011	164	126 (87.5)	24 (10.9)	10 (6.0)	4 (6.8)		
Age (years)							
< 25	72	9 (6.3)	24 (11.0)	22 (13.3)	17 (29.3)	<0.001	0.13
25–35	367	86 (59.7)	137 (62.8)	108 (65.5)	36 (62.1)		
> 35	146	49 (34.0)	57 (26.2)	35 (21.2)	5 (8.6)		
CD4 count (cells/mm^3^)							
< 200	58	16 (11.9)	18 (8.6)	18 (11.2)	6 (11.3)	0.18	0.58
200–350	101	19 (14.2)	33 (15.8)	40 (24.8)	9 (17.0)		
> 350	398	99 (73.9)	158 (75.6)	103 (64.0)	38 (71.7)		
Parity							
Multip with PPTD	7	4 (2.8)	1 (0.45)	1 (0.6)	1 (1.7)	<0.001	<0.001
Multip without PPTD	107	47 (32.9)	35 (15.9)	22 (13.3)	3 (5.1)		
Nulliparous	474	92 (63.3)	184 (83.6)	143 (86.1)	55 (93.2)		
Hepatitis C							
Negative	557	135 (93.8)	201 (91.4)	163 (98.2)	58 (98.3)	0.015	0.40
Positive	32	9 (6.25)	19 (8.64)	3 (1.70)	1 (1.69)		
Race							
Black	379	103 (79.2)	130 (62.8)	108 (65.9)	38 (64.4)	<0.001	0.006
Caucasian	140	20 (15.4)	58 (28.0)	42 (25.6)	20 (33.9)		
Other	41	7 (5.40)	19 (9.2)	14 (8.54)	1 (1.70)		

a Chi-square test across all categories

b Chi-square test comparing only boosted to unbooted PIs.

There were significant differences in the use of boosted versus unboosted PIs according to study time period, parity and ethnicity. The use of boosted PIs increased in the period from 2007–2011 as compared to 1997–2006 (87.5% vs. 12.5%, *p*<0.001) with a corresponding decrease in the use of unboosted PIs during that time period (89.1% vs. 10.9%, *p*<0.001). A larger proportion of nulliparous women were treated with unboosted versus boosted PIs (83.6% vs. 63.3%, *p*<0.001), and a larger proportion of Caucasian women received unboosted versus boosted PIs (28.0 vs. 15.4%, *p*=0.007).

### Risk of PTB

The risk of PTB according to maternal characteristics is summarized in Table 2. The overall risk of PTB in our cohort was 13.5%. In the unadjusted analysis, the use of boosted PI regimens was associated with a significantly increased risk of PTB compared to non-boosted PI regimens (OR 2.01, 95% CI 1.02–3.97), while no increased risk was observed with the use of NNRTI/NRTI-based regimens as compared to non-boosted PI-based therapy (OR 0.81, 95% CI 0.40–1.66). The highest risk of PTB was seen among women receiving no ARV treatment when compared to unboosted PIs (OR 2.70, 95% CI 1.20–6.09). Among boosted PIs, the highest risk of PTB was among ATZ/r-treated women (*n*=20) (30%), followed by LPV/r (*n*=91) (18.7%), with no cases of PTB seen among TPV/r (*n*=4) or FPV/r (*n*=4) treated women. These differences were not statistically significant (*p*=0.33). Among unboosted PIs, the highest risk of PTB was among NFV-treated women (*n*=161) (11.8%), followed by IDV (*n*=18) (11.1%) and SQV (*n*=16) (6.3%). These differences were not statistically significant (*p*=0.83). Among NRTI backbone combinations used, there was no increased risk of PTB when comparing 3TC-, ddI- or d4T-based regimens to AZT monotherapy. Other significant risk factors associated with PTB on univariate analysis included parity [multiparous with a history of previous pre-term delivery (PPTD) vs. nulliparous, OR: 15.19, 95% CI 1.85–125.8], and hepatitis C co-infection (OR: 3.20, 95% CI 1.25–8.19).

**Table 2:** Maternal characteristics and risk of pre-term delivery

Predictor variable	Pre-term *N* (%)	Term *N* (%)	Unadjusted OR	*p*	Adjusted OR	*p*
Total	71 (13.5)	454 (86.5)				
Year of delivery						
1988–1996	17 (16.8)	84 (83.2)	1.05 (0.48–1.48)	0.9		
1997–2006	34 (11.6)	259 (88.4)	0.73 (0.38–1.38)	0.33		
2007–2011	20 (15.3)	111 (84.7)	1			
Age (years)						
> 35	15 (11.8)	112 (88.2)	0.91 (0.35–1.63)	0.84	0.71 (0.27–1.87)	0.49
25–35	47 (14.2)	285 (85.8)	1.09 (0.48–1.52)	0.82	0.98 (0.45–2.15)	0.97
< 25	9 (13.8)	56 (86.2)	1		1	
ART treatment						
No treatment	13 (25.0)	39 (75.0)	2.70 (1.20–6.09)	0.017	1.50 (0.33–6.78)	0.60
NRTI/NNRTI	14 (8.8)	145 (91.2)	0.81 (0.40–1.66)	0.56	0.67 (0.27–1.63)	0.37
Boosted	23 (19.3)	96 (80.7)	2.01 (1.02–3.97)	0.045	2.17 (1.05–4.51)	0.038
Unboosted	21 (10.8)	174 (89.2)	1		1	
NRTI backbone						
None	13 (25.5)	38 (74.5)	2.59 (1.04–6.42)	0.041	1.40 (0.26–7.59)	0.69
AZT/3TC	37 (11.5)	285 (88.5)	1.12^a^ (0.53–2.35)	0.44	0.50 (0.18–1.36)	0.17
Tdf/3TC	4 (33.3)	8 (66.7)				
ABC/3TC	5 (20.0)	20 (80.0)				
D4T base	1 (8.3)	11 (91.7)	0.42^b^ (0.05–3.67)	0.77	0.20 (0.02–1.86)	0.16
DDI base	0 (0.0)	8 (100.0)				
Other	1 (25.0)	3 (75.0)	2.67 (0.21–38.56)	0.44	1.59 (0.08–33.3)	0.77
AZT	9 (11.7)	68 (88.3)	1			
CD4 count (cells/mm^3^)						
< 200	6 (11.1)	48 (88.9)	0.68 (0.27–1.73)	0.43	0.59 (0.22–1.57)	0.29
200–350	8 (8.3)	88 (91.7)	0.50 (0.24–1.07)	0.07	0.56 (0.26–1.19)	0.13
> 350	57 (15.2)	318 (84.8)	1		1	
Parity						
Multip with PPTD	3 (75.0)	1 (25.0)	15.19 (1.85–125.8)	0.012	13.28 (1.89–93.5)	0.009
Multip without PPTD	16 (17.4)	76 (82.6)	1.64 (0.86–3.04)	0.12	1.56 (0.81–3.01)	0.18
Nulliparous	52 (12.1)	377 (87.9)	1		1	
Hepatitis C						
Positive	7 (33.3)	14 (66.7)	3.20 (1.25–8.19)	0.015	4.66 (1.71–12.75)	0.003
Negative	64 (12.7)	440 (87.3)	1		1	
Race						
Black	48 (13.3)	312 (86.7)	1.01 (0.54–1.87)	0.40	1.49 (0.71–3.15)	0.29
Other	7 (18.4)	31 (81.6)	1.49 (0.59–3.79)	0.97	3.27 (1.16–9.16)	0.025
Caucasian	16 (12.6)	111 (87.4)	1		1	

a Combined OR for AZT/3TC, Tdf/3TC, ABC/3TC backbones

b Combined OR for d4T- and DDI-based backbones.

The multivariable analysis was restricted to only those women with complete data on all variables assessed, and after excluding those women with missing data on the following variables (age=4, delivery CD4 count=23, ethnicity=35 and parity=2), this analysis was conducted on 525 of the 589 patients. The association between type of PI (boosted vs. unboosted) and risk of PTB remained significant after adjusting for maternal age, delivery CD4 count, parity, hepatitis C status and ethnicity (aOR 2.17, 95% CI 1.02–4.51). Other significant risk factors remained parity (multiparous with a history of PTB vs. nulliparous, aOR 13.28, 95% CI 1.89–93.5) and hepatitis C co-infection (OR: 4.66, aOR 1.71–12.75).

## Discussion

In this single centre retrospective study, we identified a number of risk factors for PTB among HIV-positive women delivering in Canada, including type of antiretroviral therapy (ART), previous history of PTB, and hepatitis C co-infection. While untreated HIV infection is a well-known risk factor for PTB [24], among women treated with ART, we found an increased risk of PTB among those who received boosted versus unboosted PI-based regimens during pregnancy (aOR 2.04, 95% CI 1.02–4.14). Our findings of increased PTB associated with RTV boosting are consistent emerging findings from both developed and developing world settings. A study by the Agence Nationale de Recherche sur le Sida (ANRS) of France showed an increased rate of PTB among women treated with boosted versus unboosted PI (aHR 2.03, 95% CI 1.06–3.89), more specifically with respect to spontaneous delivery [25]. Our PTB rate of 19.3% among women treated with boosted PIs is also similar to that found in the Mma Bana study (21.4%) from Botswana, where all women in the PI treated arm received LPV-r during pregnancy [26]. In light of previous conflicting reports between North American and European cohorts, the concordance of our findings to the French perinatal cohort is reassuring.

In the present study, the use of unboosted PIs was not associated with an increased risk of PTB compared to NRTI- or NNRTI-based regimens. Interestingly, in a subset of previous studies (including one from our own centre) in which the type of PI used was reported, no association between PI use and PTB was seen in studies where NFV was reported as the primary PI used [7,27–29], while those in which LPV/r was used preferentially have demonstrated an increased risk of PTB [9,25,27]. We hypothesize that in the majority of studies conducted prior to 2007, unboosted PIs may have been used preferentially, thereby explaining the lack of association seen with PIs and PTB in these earlier studies. Subsequent attempts at pooling the data, considering boosted and unboosted PIs together, may have contributed to the conflicting results.

While the mechanisms underlying the potential association between RTV boosting and PTB have yet to be fully understood, there is increasing evidence that maternal and fetal adrenal dysfunction from RTV exposure may play a role in the initiation of PTB. Progesterone is a sex hormone essential for the maintenance of pregnancy, and in a mouse pregnancy model, mice exposed to PIs during pregnancy were shown to have lower progesterone levels, more pregnancy loss, less viable pups per litter, and lower fetal and placental weights compared to mice exposed only to double NRTI therapy; among the PIs studied, RTV had the strongest inhibitory effect on progesterone levels in the mouse model [30]. Lower levels of progesterone among RTV-treated women, something not assessed in the present study, may in part explain the higher rate of PTB among them. Moreover, transient fetal adrenal dysfunction from post-natal RTV exposure has been reported in a cohort of HIV-exposed uninfected newborns, with increased 17-hydroxy progesterone and dehydroepiandrosterone sulfate (DHEA-S) concentrations among newborns who received LPV-r prophylaxis compared to newborn treated with other ARV agents in the neonatal period [23]. Given that fetal signals coming from the hypothalamic–pituitary–adrenal axis play a fundamental role in the initiation of spontaneous labour [31], we suspect that transient fetal adrenal dysfunction from *in utero* RTV exposure may further contribute to the initiation of PTB.

The major limitations of our study are its retrospective nature, and our inability to adjust for other potential confounders. Due to the changing nature of the HIV epidemiology in our cohort over time, there were significant differences in maternal characteristics according to the type of PI treatment received. Nulliparous women were more likely to have received unboosted PIs, possibly reflecting first pregnancies among the first wave of prenatally infected women to have been followed and treated with ARVs. Caucasian women were also more likely to have received unboosted rather than boosted PIs, likely reflecting the increasing number of women diagnosed with HIV through immigration, compared to IV drug use or blood transfusion [32,33]. While these were adjusted for in the multivariable analysis, we were unable to adjust for maternal weight gain during pregnancy, hard drug and alcohol use, and smoking. We were also unable to control for the timing of ARV initiation, which has been reported to influence PTB [1,34,35]. Finally, we do not have information on mode of delivery, and of what proportion of pre-term births were iatrogenic caesarean section versus spontaneous PTB, which is an important consideration in the aetiology of PTB.

Given these limitations, our findings of increased risk of PTB among women treated with boosted PIs should be interpreted with caution. The success of cART in reducing mother-to-child transmission rates is a tremendous achievement, and cART remains the cornerstone of prevention. Nonetheless, in light of increased morbidity and mortality from prematurity among HIV-exposed infants [36], and the availability of alternatives to RTV-boosted PIs in pregnancy, further study is necessary to understand the role of RTV boosting, and to identify the safest most effective cART regimens for HIV-positive women.

## References

[1] European Collaborative Study; Swiss Mother and Child HIV Cohort Study (2000). Combination antiretroviral therapy and duration of pregnancy. AIDS.

[2] Thorne C, Patel D, Newell ML (2004). Increased risk of adverse pregnancy outcomes in HIV-infected women treated with highly active antiretroviral therapy in Europe. AIDS.

[3] Townsend CL, Cortina-Borja M, Peckham CS, Tookey PA (2007). Antiretroviral therapy and premature delivery in diagnosed HIV-infected women in the United Kingdom and Ireland. AIDS.

[4] Grosch-Woerner I, Puch K, Maier RF, Niehues T, Notheis G, Patel D (2008). Increased rate of prematurity associated with antenatal antiretroviral therapy in a German/Austrian cohort of HIV-1-infected women. HIV Med.

[5] Tuomala RE, Watts DH, Li D, Vajaranant M, Pitt J, Hammill H (2005). Improved obstetric outcomes and few maternal toxicities are associated with antiretroviral therapy, including highly active antiretroviral therapy during pregnancy. J Acquir Immune Defic Syndr.

[6] Dola CP, Khan R, DeNicola N, Amirgholami M, Benjamin T, Bhuiyan A (2011). Combination antiretroviral therapy with protease inhibitors in HIV-infected pregnancy. J Perinat Med.

[7] Patel K, Shapiro DE, Brogly SB, Livingston EG, Stek AM, Bardeguez AD (2010). Prenatal protease inhibitor use and risk of preterm birth among HIV-infected women initiating antiretroviral drugs during pregnancy. J Infect Dis.

[8] Cotter AM, Garcia AG, Duthely ML, Luke B, O'Sullivan MJ (2006). Is antiretroviral therapy during pregnancy associated with an increased risk of preterm delivery, low birth weight, or stillbirth?. J Infect Dis.

[9] Watts DH, Williams PL, Kacanek D, Griner R, Rich K, Hazra R (2013). Combination antiretroviral use and preterm birth. J Infect Dis.

[10] Kourtis AP, Schmid CH, Jamieson DJ, Lau J (2007). Use of antiretroviral therapy in pregnant HIV-infected women and the risk of premature delivery: a meta-analysis. AIDS.

[11] Van der Merwe K, Hoffman R, Black V, Chersich M, Coovadia A, Rees H (2011). Birth outcomes in South African women receiving highly active antiretroviral therapy: a retrospective observational study. J Int AIDS Soc.

[12] Machado ES, Hofer CB, Costa TT, Nogueira SA, Oliveira RH, Abreu TF (2009). Pregnancy outcome in women infected with HIV-1 receiving combination antiretroviral therapy before versus after conception. Sex Transm Infect.

[13] Ravizza M, Martinelli P, Bucceri A, Fiore S, Alberico S, Tamburrini E (2007). Treatment with protease inhibitors and coinfection with hepatitis C virus are independent predictors of preterm delivery in HIV-infected pregnant women. J Infect Dis.

[14] Panel on Treatment of HIV-Infected Pregnant Women and Prevention of Perinatal Transmission (2012). Recommendations for use of antiretroviral drugs in pregnant HIV-1-infected women for maternal health and interventions to reduce perinatal HIV transmission in the United States. http://aidsinfo.nih.gov/contentfiles/lvguidelines/PerinatalGL.pdf.

[15] Public Health Service Taskforce: Recommendations for Use of Antiretroviral Drugs in Pregnant HIV-1 Infected Women for Maternal Health and Interventions to Reduce Perinatal HIV-1 Transmission in the United States (2006). http://aidsinfo.nih.gov/contentfiles/PerinatalGL.

[16] DHHS Perinatal Panel Notice on Nelfinavir FDA-Pfizer Letter (2007). http://aidsinfo.nih.gov/contentfiles/upload/PeriNFVNotice1.pdf.

[17] Public Health Service Task Force: Recommendations for Use of Antiretroviral Drugs in Pregnant HIV-1-Infected Women for Maternal Health and Interventions to Reduce Perinatal HIV Transmission in the United States (2007). http://aidsinfo.nih.gov/ContentFiles/PerinatalGL02112007901.pdf.

[18] Public Health Service Task Force: Recommendations for Use of Antiretroviral Drugs in Pregnant HIV-1-Infected Women for Maternal Health and Interventions to Reduce Perinatal HIV Transmission in the United States (2008). http://aidsinfo.nih.gov/contentfiles/PerinatalGL001020.pdf.

[19] Baroncelli S, Tamburrini E, Ravizza M, Dalzero S, Tibaldi C, Ferrazzi E (2009). Antiviral treatment in pregnancy: a six-year prospective on recent trends in prescription patterns, viral load suppression, and pregnancy outcomes. AIDS Patient Care STDS.

[20] Andany N, Loutfy MR (2013). HIV protease inhibitors in pregnancy: pharmacology and clinical use. Drugs.

[21] Granfors MT, Wang JS, Kajosaari LI, Laitila J, Neuvonen PJ, Backman JT (2006). Differential inhibition of cytochrome P450 3A4, 3A5 and 3A7 by five human immunodeficiency virus (HIV) protease inhibitors in vitro. Basic Clin Pharmacol Toxicol.

[22] Beshay VE, Carr BR, Rainey WE (2007). The human fetal adrenal gland, corticotropin-releasing hormone, and parturition. Semin Reprod Med.

[23] Simon A, Warszawski J, Kariyawasam D, Le Chenadec J, Benhammou V, Czernichow P (2011). Association of prenatal and postnatal exposure to V-ritonavir and adrenal dysfunction among uninfected infants of HIV-infected mothers. JAMA.

[24] Lopez M, Figueras D, Hernandez S, Lonca M, Garcia R, Palacio M (2012). Association of HIV infection with spontanous and iatrogneic preterm delivery. AIDS.

[25] Sibiude J, Warszawski J, Tubiana R, Dollfus C, Faye A, Rouzioux C (2012). Premature delivery in HIV-infected women starting protease inhibitor therapy during pregnancy: role of the ritonavir boost?. Clin Infect Dis.

[26] Powis KM, Kitch D, Ogwu A, Hughes MD, Lockman S, Leidner J (2011). Increased risk of preterm delivery among HIV-infected women randomized to protease versus nucleoside reverse transcriptase inhibitor-based CART during pregnancy. J Infect Dis.

[27] Carceller A, Ferreira EM, Alloul S, Lapointe N (2009). Lack of effect on prematurity, birth weight and infant growth from exposure to protease inhibitors in utero and after birth. Pharmacotherapy.

[28] Morris AB, Cu-Uvin S, Harwekk JI, Garb J, Zorrilla C, Vajaranant M (2000). Multicenter review of protease inhibitors in 89 pregnancies. J Aquir Immune Defic Syndr.

[29] Schulte J, Dominguez K, Sukalac T, Bohannon B, Fowler MG, Pediatric Spectrum of HIV Disease Consortium (2007). Declines in low birth weight and preterm birth among infants who were born to HIV-infected women during an era of increased use of maternal antiretroviral drugs: Pediatric Spectrum of HIV Disease, 1989–2004. Pediatrics.

[30] Papp E, Mohammadi H, Loutfy MR, Yudin MH, Murphy KE, Walmsley SL (2015). HIV protease inhibitor use during pregnancy is associated with decreased progesterone levels, suggesting a potential mechanism contributing to fetal growth restriction. J Infec Dis.

[31] Wimalasundera RC, Larbalestier N, Smith JH, de Ruiter A, McG Thom SA (2002). Pre-eclampsia, antiretroviral therapy, and immune reconstitution. Lancet.

[32] Brophy C, Kakkar F, Sauve L, Lee T, Bitnun A, Alimenti A (2014). Transition of Youth Living with HIV from Pediatric to Adult Care in Canada: Past, Present, and Future. Canadian Journal of Infectious Diseases and Medical Microbiology.

[33] Antoniou T, Zagorski B, Macdonald EM, Bayoumi AM, Raboud J, Brophy J (2014). Trends in live births and adverse neonatal outcomes among HIV-positive women in Ontario, Canada, 2002–2009: a descriptive population-based study. Int J STD AIDS.

[34] Chen JY, Ribaudo HJ, Souda S, Parekh N, Ogwu A, Lockman S (2012). Highly active antiretroviral therapy and adverse birth outcomes among HIV-infected women in Botswana. J Infect Dis.

[35] Ezechi OC, David AN, Gab-Okafor CV, Ohwodo H, Oladele DA, Kalejaiye OO (2012). Incidence of and socio-biologic risk factors for spontaneous preterm birth in HIV positive Nigerian women. BMC Pregnancy Childbirth.

[36] Warszawski J, Le Chenadec J, Mandelbrot L, Faye A, Dolfus X, Tubiana R Infant mortality among HIV-uninfected children during the HAART era in France – ANRS French perinatal cohort.

